# En-bloc kidney transplants from very small pediatric donors: a propensity score matched analysis

**DOI:** 10.3389/fped.2025.1570489

**Published:** 2025-04-25

**Authors:** Silvia Oberparleiter, Felix J. Krendl, Thomas Resch, Rupert Oberhuber, Hannah Esser, Florian Ponholzer, Annemarie Weissenbacher, Robert Breitkopf, Hannes Neuwirt, Stefan Schneeberger, Manuel Maglione, Benno Cardini

**Affiliations:** ^1^ Department of Visceral, Transplant and Thoracic Surgery, Center of Operative Medicine, Medical University of Innsbruck, Innsbruck, Austria; ^2^ Department of Anesthesiology and Intensive Care, Medical University of Innsbruck, Innsbruck, Austria; ^3^ Department of Internal Medicine IV, Nephrology and Hypertension, Medical University of Innsbruck, Innsbruck, Austria

**Keywords:** kidney transplantation, long-term outcome, marginal organs, postoperative complications, pediatric donors

## Abstract

**Background:**

Kidneys from brain-death small pediatric donors ≤2 years are still classified as marginal organs. Herein, we analyse the outcomes following en-bloc kidney transplantation (EBKT) from pediatric donors ≤2 years into adult recipients compared to standard criteria donor kidney transplant recipients (SKTs).

**Methods:**

A retrospective single center analysis of a prospectively collected and auditable database identified six EBKTs and 75 SKTs between January 2015 and June 2017. Propensity score matching minimized selection bias.

**Results:**

After a median follow-up of 74 months, five-year patient and graft survival were 100%, each in the EBKTs group. Following SKTs, the five-year patient survival rate was 94.7%, likewise death-censored graft survival reached 94.7%. Two EBKT cases experienced unilateral arterial graft thrombosis requiring unilateral nephrectomy, with full recovery and good kidney function. At hospital discharge, recipients of EBKTs showed decreased eGFR compared to SKTs, however, from 3 months onward this reversed and following a median follow-up of 74 months the median eGFR was twice as high after EBKT compared to SKT (107 ml/min/1.73m^2^ vs. 52 ml/min/1.73m^2^, *p* < 0.001). These favourable results persist in the PSM analysis.

**Conclusion:**

EBKTs from very small pediatric donors show excellent long-term kidney function. The higher incidence of postoperative complications does not translate into poorer mid-term patient and graft survival.

## 1 Introduction

Kidney transplantation (KT) represents the therapy of choice for patients suffering from end-stage-renal disease with need for dialysis (1). Compared to dialysis, kidney transplant recipients benefit from improved quality of live and a significantly lower risk of mortality and cardiovascular events (2–4). Additionally, kidney transplantation is associated with considerable cost-effectiveness advantages over chronic dialysis (5). However, to date the transplant community faces a large discrepancy between the number of patients on the waiting list and the availability of suitable donor organs, leading to ever increasing waiting lists (6, 7). By the end of 2023, within the Eurotransplant (ET) network, 10,404 patients were actively listed for a kidney transplant. A total of 3,161 kidney transplants from deceased donors and 1,323 from living donors were performed while 5,943 new registrations were recorded. 935 patients had to be removed from the waiting list due to death or being deemed unfit for transplantation. The median waiting time for a kidney transplant in the ET region in 2023 was 55 months (7). Hence strategies to enhance the donor pool have been pursued over the last years to counteract this discrepancy. For instance, programs such as the Eurotransplant (ET) senior program in the ET region have been shown to shorten waiting-times as well as to increase the donor pool without comprising outcomes (8). Moreover, hypothermic and normothermic machine perfusion have gained increasing interest over the last decade and further improved utilization rates (9). Despite general efforts to increase the donor pool, reluctancy still exists when it comes to accepting kidney grafts from small pediatric donors for adult recipients. Technical difficulties, higher surgical complication rates, a possible inadequate nephron mass, an increased incidence of acute rejection episodes, and worse graft survival rates have been cited as key factors resulting in the under-utilization of these organs (10–12). Suneja et al. reported utilization rates of small pediatric donor kidneys between 2005 and 2014 sobering. Utilization reached only 53% for donors weighing between 10 and 14.9 kg (62.7% en-bloc KT, 37.3% split kidneys), and even less in smaller donors with 32% for those between 5 and 9.9 kg (84.4% en-bloc KT, 15.6% split kidneys) and 16% for donors weighing 0–4.9 kg (98.7% en-bloc KT, 2.3% split kidneys) (13). However, improvements in peri- and postoperative care, as well as improved immunological management have shifted this perspective in recent years (14–16).

In the present study we aimed to analyse perioperative morbidity as well as (long-term) outcomes following en-bloc kidney transplantation (EBKTs) from pediatric donors younger than 24 months of age into adult recipients and compared the results of EBKTs with those of recipients of standard criteria donor kidney grafts (SKT) performed at our center during the same period. The primary endpoints were 5-year graft and patient survival rate. Secondary outcomes included perioperative morbidity such as vascular, ureteral, and immunological complications.

## 2 Material and methods

In this retrospective single center analysis consecutive first or second-transplant recipients undergoing EBKT from pediatric, brain death (DBD) donors younger than 24 months of age between January 2015 and June 2017 were included. Adult recipients of first or second kidney transplants receiving SKTs in the same period served as controls.

Exclusion criteria were recipient age less than 18 years, combined transplantations as well as living-donor KT.

In total 81 patients were included, 6 in the EBKT group and 75 in the SKT group. The data were retrospectively analysed from a prospectively collected and auditable medical database. The study was approved by the local ethics committee (EC-number 1037/2018) and results are reported according to the STROBE guidelines (17).

### 2.1 Surgical procedure

EBKT was performed as described previously (18–20). In brief, the cranial aorta and vena cava were closed via a running suture with 6-0 PDS. Lumbar branches were ligated using 4-0 vicryl. Access to the external iliac artery (EIA) and external iliac vein (EIV) was gained through a Gibson incision; the same incision as for SKT. Both, the donor's vena cava and aorta were anastomosed in an end-to-side fashion to the recipients' EIV and EIA using 5-0 prolene. The ureterocystostomy was performed using Lich-Gregoire ureteral implantation technique. Both ureters were stented separately using double J stents.

### 2.2 Perioperative care

Standard immunosuppression consisted of induction therapy with 20 mg of basiliximab (day 0 and 3, Simulect®; Novartis, Dublin, Ireland) for all first kidney transplant recipients and a single dose of antithymocyte globuline (8 mg/kg, at day of transplantation, (ATG-Fresenius®; Fresenius Biotech, Gräfelfing, Germany) for all second kidney transplant recipients. Maintenance therapy consisted of tacrolimus (trough levels of 8–10 ng/ml during first three months, Advagraf®; Astellas Pharma, Vienna, Austria), mycophenolate mofetil (twice daily 1,000 mg, CellCept®; Roche Austria, Vienna, Austria) and tapered steroids according to institutional standard. Antimicrobial prophylaxis consisted of ampicillin/sulbactam single shot (Pfizer®; Vienna, Austria). Every patient received trimethoprim/sulfamethoxazole (Eusaprim®; Aspen Pharma Trading Limited, Dublin, Ireland three times a week for pneumocystis jirovecii prophylaxis. In case of CMV seropositive donor and/or recipient (D+/R−, D+/R+ or D−/R+), antiviral prophylaxis with valganciclovir (Valcyte®; Roche Austria) was administered for 90 days.

For patients receiving EBKT, postoperative management additional consisted of 6-hourly doppler ultrasound control, intravenous heparinisation for the first three days with a target aPTT of 40–45 s and maintenance of systolic blood pressure at approximately 100 mmHg. Blood pressure adjustments were made based on the donor's age, considering physiologic blood pressure ranges in infants and toddlers (21). Delayed graft function (DGF) was defined as the need for dialysis within the first postoperative week except for dialysis for hyperkalaemia or hypervolemia within the first 12 h posttransplant (22).

### 2.3 Recipient selection

Recipient selection criteria for EBKT lack specific guidelines. Due to the type of organ offer (center offer for EBKT-donor organs) we selected recipients with none or controlled hypertension, as well as a BMI less than 30 kg/m^2^ and a low anaesthesiologic risk by means of cardiopulmonary comorbidities.

**Figure 1:**
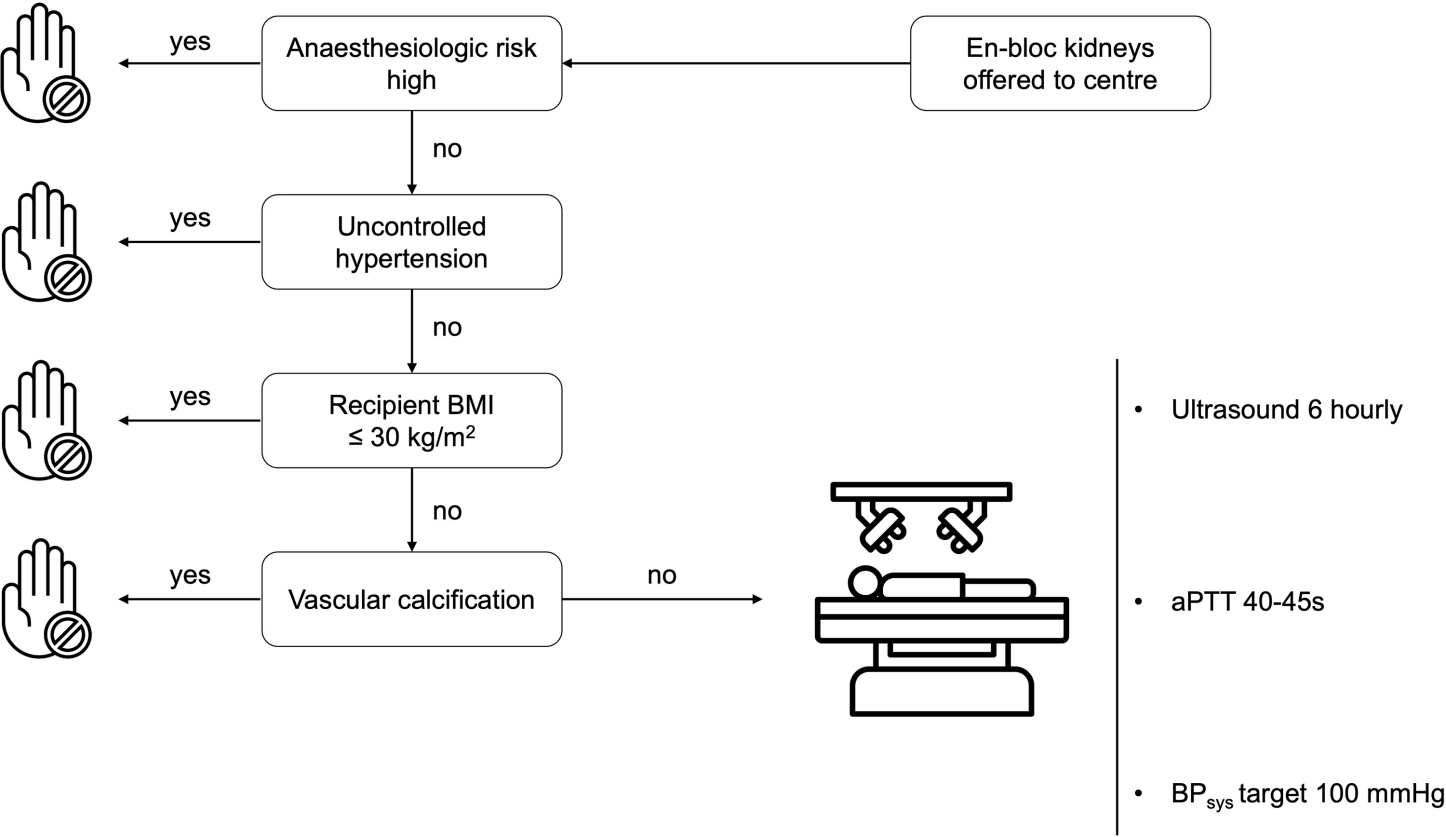
Algorithm for recipient selection and postoperative surveillance.

Patients with uncontrolled hypertension or vascular calcifications were not considered for EBKT. Informed consent for receiving en-bloc pediatric grafts was obtained prior to transplantation (Algorithm in Figure 1).

### 2.4 Statistical analysis

Data are presented as mean (SD), median (range) or numbers with percentages as appropriate. Differences between control and study group were tested using the independent-sample Mann–Whitney *U* test for non-normally distributed continuous variables, two-tailed student's *t*-test for normally distributed continuous variables and Fisher's exact test for categorical variables. Patient and graft survival rates were analysed using Kaplan–Meier estimator and log-rank test. Propensity score matched (PSM) analysis was performed with a matching ratio of 1:10 and a caliper of 1.622 (0.2 × [standard deviation of ln(propensity score)] with the variables: recipient BMI and number of antihypertensive medications prior to kidney transplantation [based on our recipient selection algorithm (see Figure 1)]. For one EBKT recipient only one matched control was found, resulting in a PSM control cohort of 51 patients.

Two-tailed *p*-values ≤0.05 were considered statistically significant. Analyses were performed using SPSS® version 29 (IBM, Armonk, New York, USA). Course of creatinine and eGFR was displayed with GraphPad Prism 10.0.3 (GraphPad Software Inc., La Jolla, California, USA).

## 3 Results

Donor and recipient demographics are depicted in Table 1. Donor age and donor BMI differed significantly between groups. Median donor weight in the EBKT group was 8.8 kg (3–14 kg). According to our proposed recipient selection regimen, recipient BMI was lower in EBKT recipients (21.5 kg/m^2^ vs. 25.5 kg/m^2^, *p* = 0.007). Mean kidney donor risk index (KDRI) was higher in the EBKT group (1.27 vs. 1.02, *p* < 0.001).

**Table 1:** Recipient and donor demographics.

Baseline characteristics	SKT, *n* = 75	EBKT, *n* = 6	OR (95% CI)	*p*-value
Recipient age, median (range)	51 years (21–75)	45 years (21–51)		*p* = 0.086
Recipient male gender, *n* (%)	44 (58.7%)	71 (66.7%)	0.710 (0.122–4.119)	*p* = 0.528
Recipient BMI kg/m^2^, mean ± SD	25.5 ± 4.4	21.5 ± 2.4		***p*** **=** **0.007**
Number of antihypertensive medications pre-NTX, median (range)	2 (0–6)	1.5 (0–2)		*p* = 0.302
Re-TX, *n* (%)	17 (22.7%)	3 (50%)	3.412 (0.630–18,474)	*p* = 0.157
Time on dialysis, median (range)	49 months (3–211)	47 months (17–75)		*p* = 0.684
Donor age, median (range)	47 years (20–59)	10 months (1–24)		***p*** **<** **0.001**
Donor male gender, *n* (%)	51 (68%)	2 (33.3%)	4.250 (0.727–24.835)	*p* = 0.175
Donor weight, median (range)	80 kg (50–136)	8.8 kg (3.0–14.0)		***p*** **<** **0.001**
Donor BMI (kg/m^2^), mean ± SD	26.3 ± 4.1	14.3 ± 2.2		***p*** **<** **0.001**
Kidney donor risk index (KDRI), mean ± SD	1.02 ± 0.26	1.27 ± 0.10		***p*** **<** **0.001**
HLA A mismatches, mean ± SD	0.79 ± 0.64	1.0 ± 0.63		*p* = 0.458
0 and 1	66 (88%)	5 (83.3%)		
2	9 (12%)	1 (16.7%)		
HLA B mismatches, mean ± SD	1.08 ± 0.61	1.17 ± 0.75		*p* = 0.793
0 and 1	58 (77.3%)	4 (66.7%)		
2	17 (22.7%)	2 (33.3%)		
HLA DR mismatches, mean ± SD	0.97 ± 0.64	0.83 ± 0.98		*p* = 0.745
0 and 1	61 (81.3%)	4 (66.7%)		
2	14 (18.7%)	2 (33.3%)		
Total (HLA-A, HLA-B and HLA-DR) mismatches, mean ± SD	2.84 ± 1.17	3.0 ± 1.79		*p* = 0.837
Cause of end stage renal disease				
Glomerulonephritis	38 (46.9%)	2 (33.3%)		
Diabetic nephropathy	7 (8.6%)	0		
Hereditary renal disease	13 (16.0%)	2 (33.3%)		
Vascular nephropathy	8 (9.9%)	0		
Others	15 (18.5%)	2 (33.3%)		
Moderate to high risk of disease recurrence, *n* (%)	27 (36.0%)	1 (16.67%)	0.786 (0.122–5.041)	*p* = 1.0

BMI, body mass index; SD, standard deviation; TX, transplantation; HLA, human leukocyte antigen. Moderate to high risk of disease recurrence included patients with IgA nephropathy, lupus nephritis, focal segmental glomerulosclerosis, atypical hemolytic uremic syndrome, and membranoproliferative glomerulonephritis.

Values in bold indicate statistical significance (*p* < 0.05).

### 3.1 Peri- and postoperative course

Induction and maintenance immunosuppression therapy was similar between groups. Cold ischemia time as well as anastomosis time were comparable between groups (see Table 2). The postoperative course was uneventful in two out of six patients with immediate good graft function. Regarding 90-day postoperative complication rates, the relaparotomy rate was higher in EBKTs at 66.7% (4/6) compared to 14.7% (11/75) in SKTs (*p* = 0.010). Vascular thrombosis occurred in 1/75 (1.3%) of SKTs compared to 2/6 (33.3%) of EBKTs resulting in a relative risk for graft thrombosis following EBKT of 1.48 (95% CI: 0.840–2.608), OR 37 (95%CI: 2.740–499.545), *p* = 0.013. However, the wide confidence interval reflects the small sample size, necessitating cautious interpretation. In both cases, graft thrombosis occurred on postoperative day 2 and led to unilateral transplant nephrectomy. One of the two EBKTs recipients who developed graft thrombosis never reached the targeted aPTT of 40–45 s during first postoperative days. The other recipient received the smallest en-bloc kidneys in our series from an infant donor weighing three kilograms. In both patients, the medially located kidney was affected from graft thrombosis. Of note, both EBKT recipients who developed graft thrombosis had undergone kidney re-transplantation. Both recipients recovered well from this event and showed good kidney function at last follow-up with an eGFR of 85 and 110 ml/min/1.73^2^, respectively. Another recipient in the EBKTs group underwent relaparotomy due to kinking of the vascular pedicle. In this case, graft perfusion was compromised for a short period. However, following repositioning homogenous reperfusion of the affected graft could be achieved and the graft therefore preserved. No ureteral complications were observed in the EBKTs group. Three patients in the SKT group developed urinary leakage, with all of them undergoing surgical treatment with ureteral reimplantation.

**Table 2:** Perioperative data and 90-day morbidity.

Perioperative characteristics	SKT, *n* = 75	EBKT, *n* = 6	OR (95% CI)	*p*-value
Basiliximab, *n* (%)	58 (77.3%)	3 (50%)	0.293 (0.054–1.587)	*p* = 0.157
ATG, *n* (%)	17 (22.7%)	3 (50%)	3.412 (0.630–18,474)	*p* = 0.157
Tacrolimus, *n* (%)	68 (90.7)	6 (100%)	0.907 (0.843–0.975)	*p* = 1.0
Cyclosporin, *n* (%)	7 (9.3)	0		*p* = 1.0
Steroid, *n* (%)	75 (100%)	6 (100%)		
MMF, *n* (%)	75 (100%)	6 (100%)		
Cold ischemia time in h, mean ± SD	13.88 ± 4.9	14.67 ± 5.0		*p* = 0.722
Anastomosis time in min, mean ± SD	29 ± 7	28 ± 6		*p* = 0.705
DGF, *n* (%)	33 (44.0%)	4 (66.7%)	2.545 (0.439–14.759)	*p* = 0.404
Thrombosis, *n* (%)	1 (1.3%)	2 (33.3%)	37.0 (2.740–499,545)	***p*** **=** **0.013**
Bleeding, *n* (%)	9 (12.0%)	1 (16.7%)	1,467 (0.153–14,015)	*p* = 0.559
Lymphocele, *n* (%)	3 (4.0%)	1 (16.7%)	4,8 (0.419–54,958)	*p* = 0.269
Ureteral complications, *n* (%)	3 (4.0%)	0		*p* = 1.0
Relaparotomy, *n* (%)	11 (14.7%)	4 (66.7%)	11.636 (1.897–71,383)	***p*** **=** **0.010**
Immunological complications, *n* (%)	3 (4.0%)	0		*p* = 1.0
Postoperative LOS, median (range)	14 days (5–57)	17.5 days (10–25)		*p* = 0.588

ATG, antithymocyte globuline; MMF, mycophenolate mofetil; DGF, delayed graft function; LOS, length of stay.

Values in bold indicate statistical significance (*p* < 0.05).

DGF occurred in 44% of SKTs compared to 66.7% of EBKTs (*p* = 0.404). No primary non-function (PNF) was observed in the EBKT group. PNF occurred in one patient in the SKT group. This patient suffered from a complex postoperative course with thrombosis of a reconstructed lower pole artery, multiple relaparotomies due to recurrent bleeding as well as infectious complications finally leading to the patients’ death on POD41.

No acute rejection episodes occurred following EBKT. Three patients in the SKT group experienced acute and/or chronic rejection, which ultimately led to graft loss in two cases (see patient and graft survival).

**Figure 2:**
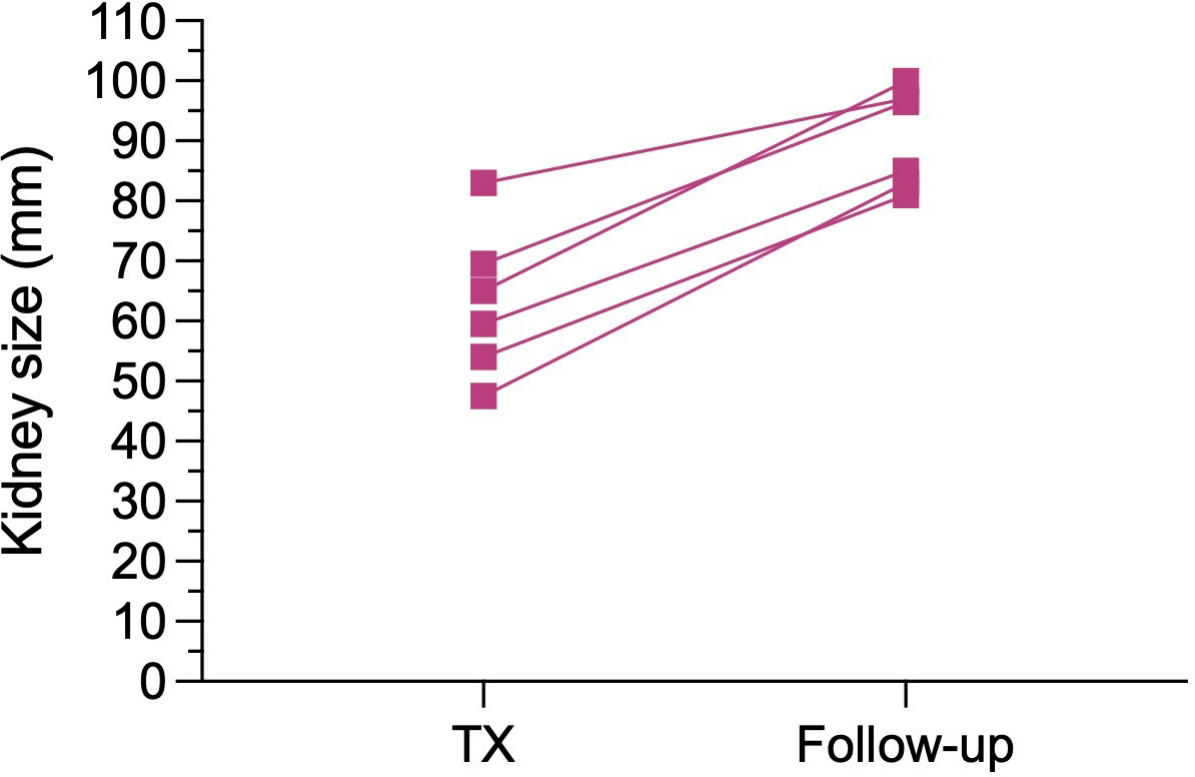
Increase in kidney size, measured during back-table preparation (i.e., TX) and by ultrasound at a median follow-up of 6.4 months postoperatively (i.e., Follow-up).

The median postoperative length of stay (LOS) was 14 days in the SKT group compared to 17.5 days in the EBKT group (*p* = 0.588). Median creatinine at hospital discharge was 2.1 mg/dl for EBKTs compared to 1.5 mg/dl for SKTs (*p* = 0.553). The corresponding estimated glomerular filtration rates (eGFR) were 35 and 43 ml/min/1.73m^2^, respectively (*p* = 0.804). Kidney size, assessed during back-table preparation as well as postoperatively using ultrasound, demonstrated a significant increase over time from 62 mm (47.5–83.0) to 91 mm (81–100) after a median follow-up of 6.4 months, indicating a size increase of 46.4% (see Figure 2).

**Figure 3:**
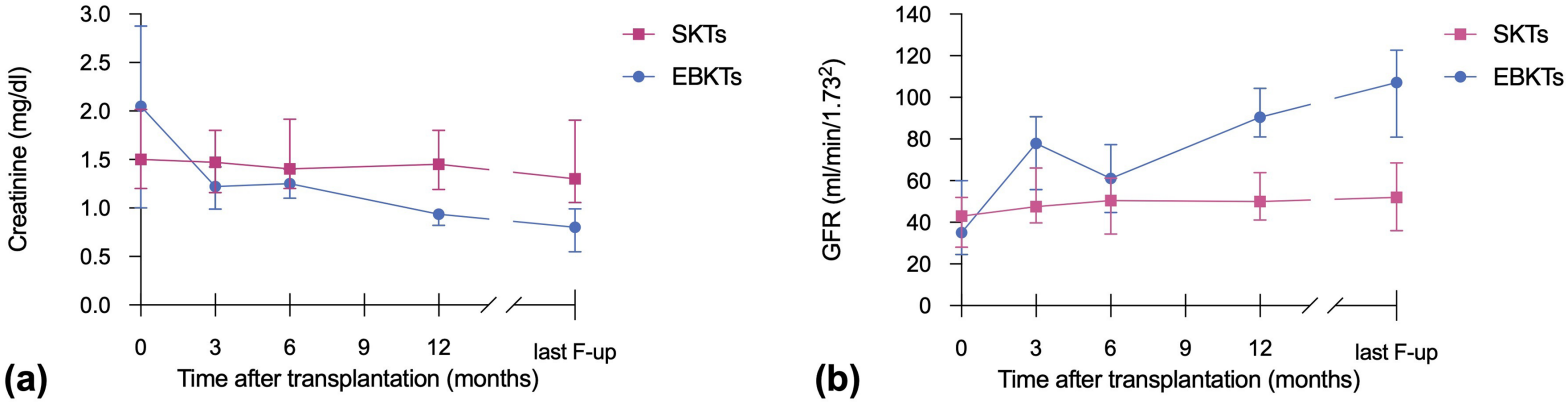
Course of **(a)** creatinine and **(b)** eGFR after SKT vs. EBKT.

At one year post transplant, patients receiving EBKTs showed significantly lower median serum creatinine levels (0.94 vs. 1.45 mg/dl, *p* = 0.002) and higher median eGFR (91 ml/min/1.73m^2^ vs. 50 ml/min/1.73m^2^, *p* = 0.001) compared to SKT recipients. These favourable results continued until last follow-up (median 74 months): median serum creatinine of 0.8 mg/dl compared to 1.3 mg/dl and median eGFR of 107 ml/min/1.73m^2^ compared to 52 ml/min/1.73m^2^ in EBKTs and SKTs, respectively (*p* = 0.001 and *p* < 0.001, respectively; see Figure 3).

### 3.2 Patient and graft survival

**Figure 4:**
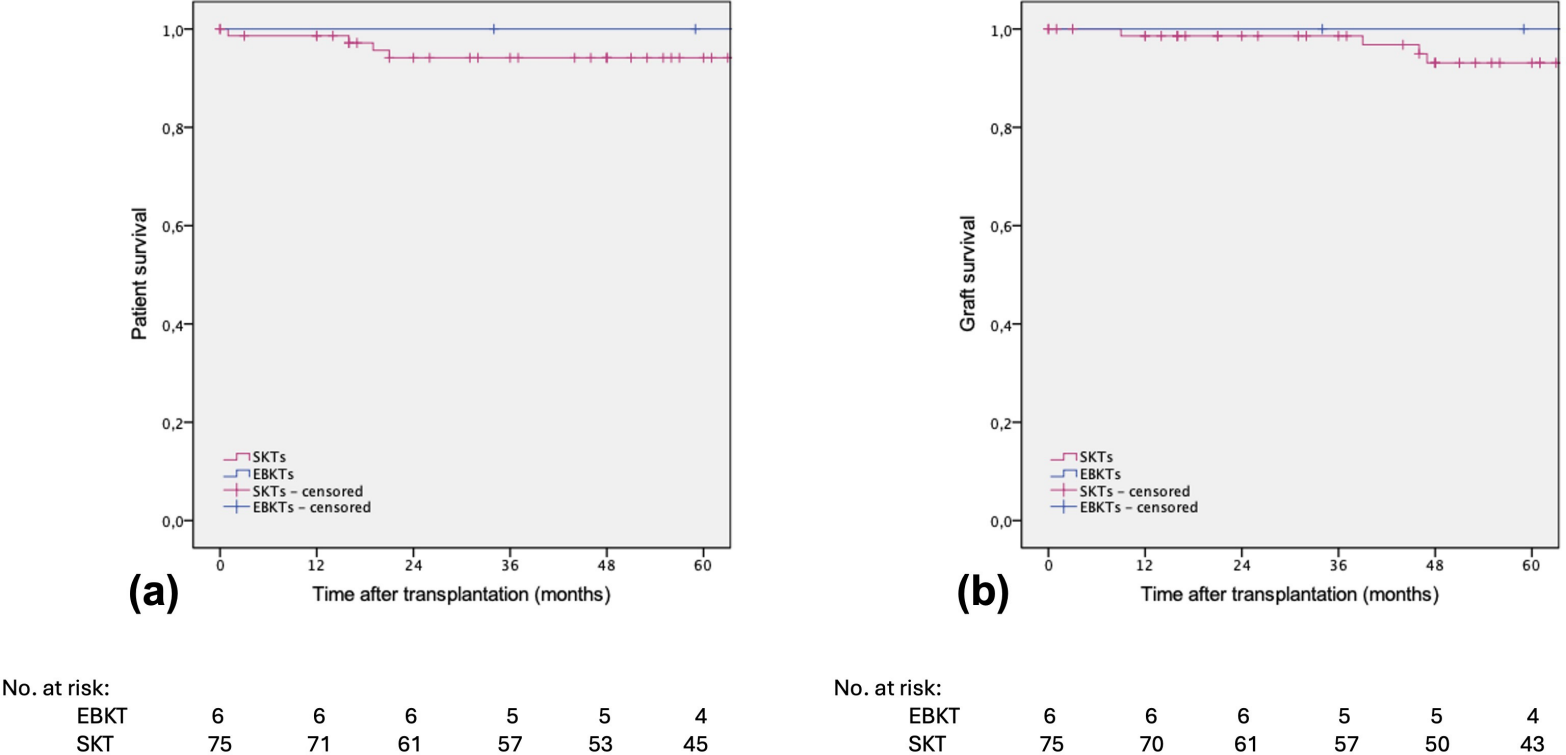
Estimated **(a)** patient survival and **(b)** death-censored graft survival after SKT vs. EBKT. *P* = 0.601 and *p* = 0.763, respectively (log rank test).

After a median follow-up of 74 months, no patient or graft loss was observed in the EBKT group. In the SKT group, four patients died during follow-up, two with a functioning graft at month 16 and 22 post transplantation. One patient died one month after nephrectomy of the non-functioning kidney graft from unknown cause. One patient, as mentioned above, died following a complex perioperative course during the initial hospital stay. Graft loss occurred in four patients in the SKT group within 5 years of follow up, one due to an acute vascular rejection type IIb according to BANFF 09 and one due to chronic rejection. Another patient experienced recurrence of IgA nephropathy, and one graft loss occurred after 3.8 years due to unknown reason (two biopsies revealed no signs of rejection), see Figure 4.

### 3.3 Propensity score matched cohort

In the PSM cohort we compared 6 EBKT recipients with 51 matched SKT recipients. Demographic and perioperative data of the PSM cohort are shown in Tables 3, 4, respectively. As in the entire cohort, the incidence of arterial thrombosis and the need for re-laparotomy were significantly higher in the EBKT group compared to the SKT group (*p* < 0.009 and *p* = 0.007, respectively).

**Table 3:** Recipient and donor demographics of the PSM cohort.

Baseline characteristics	SKT, *n* = 51	EBKT, *n* = 6	OR (95% CI)	*p*-value
Recipient age, median (range)	51 years (25–74)	45 years (21–51)		*p* = 0.128
Recipient male gender, *n* (%)	28 (54.9%)	71 (66.7%)	0.609 (0.102–3.627)	*p* = 0.686
Recipient BMI kg/m^2^, mean ± SD	23.4 ± 2.9	21.5 ± 2.4		*p* = 0.124
Number of antihypertensive medications pre-NTX, median (range)	2 (0–5)	1.5 (0–2)		*p* = 0.527
Re-TX, *n* (%)	13 (25.5%)	3 (50%)	2,923 (0.524–16.320)	*p* = 0.335
Time on dialysis, median (range)	49 months (3–88)	47 months (17–75)		*p* = 0.630
Donor age, median (range)	47 years (20–59)	10 months (1–24)		***p*** **<** **0.001**
Donor male gender, *n* (%)	36 (70.6%)	2 (33.3%)	4.8 (0.793–29.070)	*p* = 0.088
Donor weight, median (range)	80 kg (52–136)	8.8 kg (3.0–14.0)		***p*** **<** **0.001**
Donor BMI (kg/m^2^), mean ± SD	26.0 ± 3.9	14.3 ± 2.2		***p*** **<** **0.001**
Kidney donor risk index (KDRI), mean ± SD	1.01 ± 0.17	1.27 ± 0.10		***p*** **<** **0.001**
HLA A mismatches, mean ± SD	0.78 ± 0.64	1.0 ± 0.63		*p* = 0.439
0 and 1	45 (88.2%)	5 (83.3%)		
2	6 (11.8%)	1 (16.7%)		
HLA B mismatches, mean ± SD	1.02 ± 0.58	1.17 ± 0.75		*p* = 0.573
0 and 1	42 (82.4%)	4 (66.7%)		
2	9 (17.6%)	2 (33.3%)		
HLA DR mismatches, mean ± SD	0.98 ± 0.62	0.83 ± 0.98		*p* = 0.607
0 and 1	42 (82.4%)	4 (66.7%)		
2	9 (17.6%)	2 (33.3%)		
Total (HLA-A, HLA-B and HLA-DR) mismatches, mean ± SD	2.78 ± 1.08	3.0 ± 1.79		*p* = 0.670
Cause of end stage renal disease				
Glomerulonephritis	25 (49.0%)	2 (33.3%)		
Diabetic nephropathy	3 (5.9%)	0		
Hereditary renal disease	9 (17.6)	2 (33.3%)		
Vascular nephropathy	4 (7.8%)	0		
Others	10 (19.6%)	2 (33.3%)		
Moderate to high risk of disease recurrence, *n* (%)	17 (33.3%9	1 (16.67%)	0.902 (0.135–6.005)	*p* = 1.0

BMI, body mass index; SD, standard deviation; TX, transplantation; HLA, human leukocyte antigen. Moderate to high risk of disease recurrence included patients with IgA nephropathy, lupus nephritis, focal segmental glomerulosclerosis, atypical hemolytic uremic syndrome, and membranoproliferative glomerulonephritis.

Values in bold indicate statistical significance (*p* < 0.05).

**Table 4:** Perioperative data and 90-day morbidity of the PSM cohort.

Perioperative characteristics	SKT, *n* = 51	EBKT, *n* = 6	OR (95% CI)	*p*-value
Basiliximab, *n* (%)	37 (72.5%)	3 (50%)	0.378 (0.068–2.101)	*p* = 0.349
ATG, *n* (%)	14 (27.5%)	3 (50%)	2.643 (0.476–14.677)	*p* = 0.349
Tacrolimus, *n* (%)	48 (94.1)	6 (100%)	1.125 (1.024–1.236)	*p* = 1.0
Cyclosporin, *n* (%)	3 (5.9)	0		*p* = 1.0
Steroid, *n* (%)	51 (100%)	6 (100%)		
MMF, *n* (%)	51 (100%)	6 (100%)		
Cold ischemia time in h, mean ± SD	13.77 ± 5.3	14.67 ± 5.0		*p* = 0.693
Anastomosis time in min, mean ± SD	27 ± 6	28 ± 6		*p* = 0.806
DGF, *n* (%)	22 (43.1%)	4 (66.7%)	2.636 (0.442–15.720)	*p* = 0.396
Thrombosis, *n* (%)	0	2 (33.3%)		***p*** **=** **0.009**
Bleeding, *n* (%)	6 (11.8%)	1 (16.7%)	1.5 (0.149–15.109)	*p* = 0.562
Lymphocele, *n* (%)	2 (3.9%)	1 (16.7%)	4.9 (0.375–64,072)	*p* = 0.288
Ureteral complications, *n* (%)	2 (3.9%)	0		*p* = 1.0
Relaparotomy, *n* (%)	6 (11.8%)	4 (66.7%)	15.0 (2.245–100.201)	***p*** **=** **0.007**
Immunological complications, *n* (%)	2 (3.9%)	0		*p* = 1.0
Postoperative LOS, median (range)	14 days (5–42)	17.5 days (10–25)		*p* = 0.484

ATG, antithymocyte globuline; MMF, mycophenolate mofetil; DGF, delayed graft function; LOS, length of stay.

Values in bold indicate statistical significance (*p* < 0.05).

**Figure 5:**
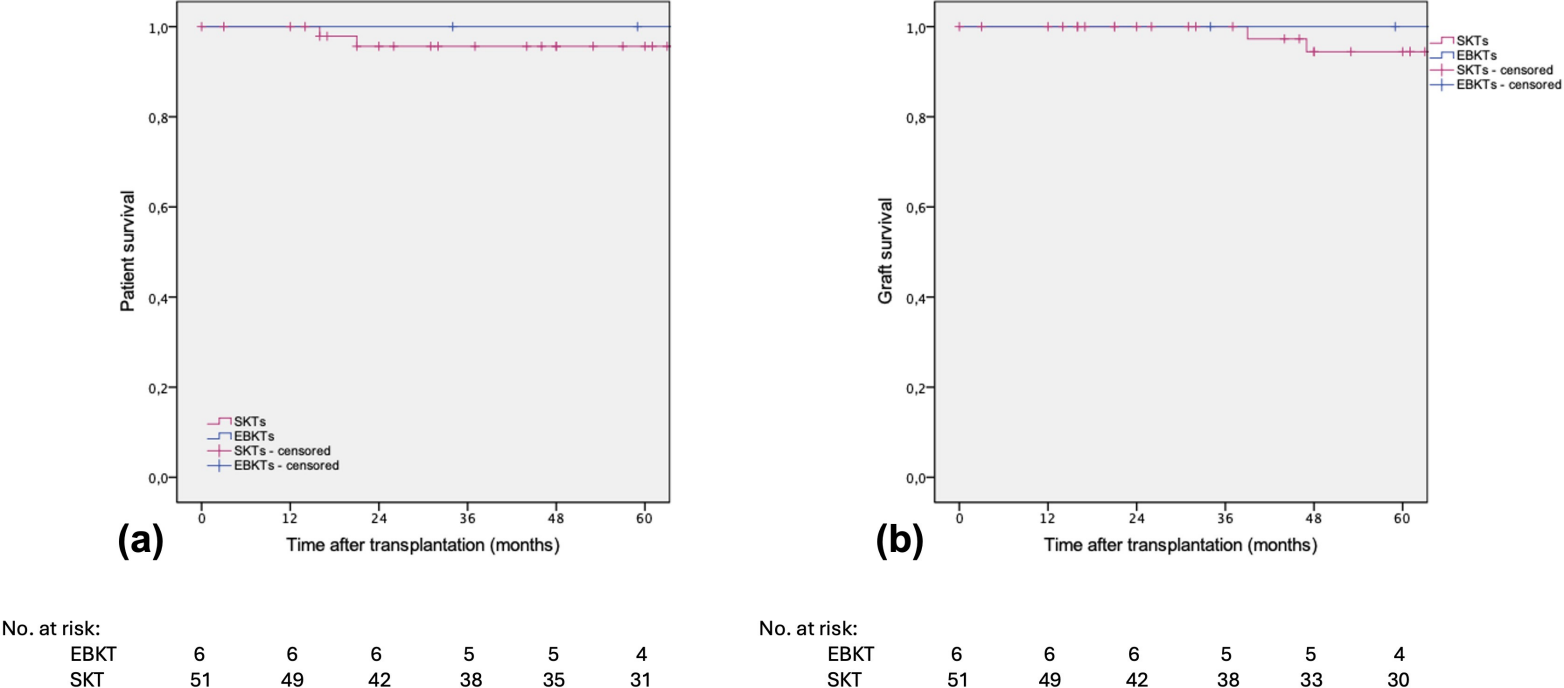
Estimated **(a)** patient survival and **(b)** death-censored graft survival after SKT vs. EBKT of the PSM cohort. *P* = 0.607 and *p* = 0.595, respectively (log rank test).

Both, patient and death-censored graft survival were 96.1% in the SKT group compared to 100% in the EBKT group (Figure 5).

**Figure 6:**
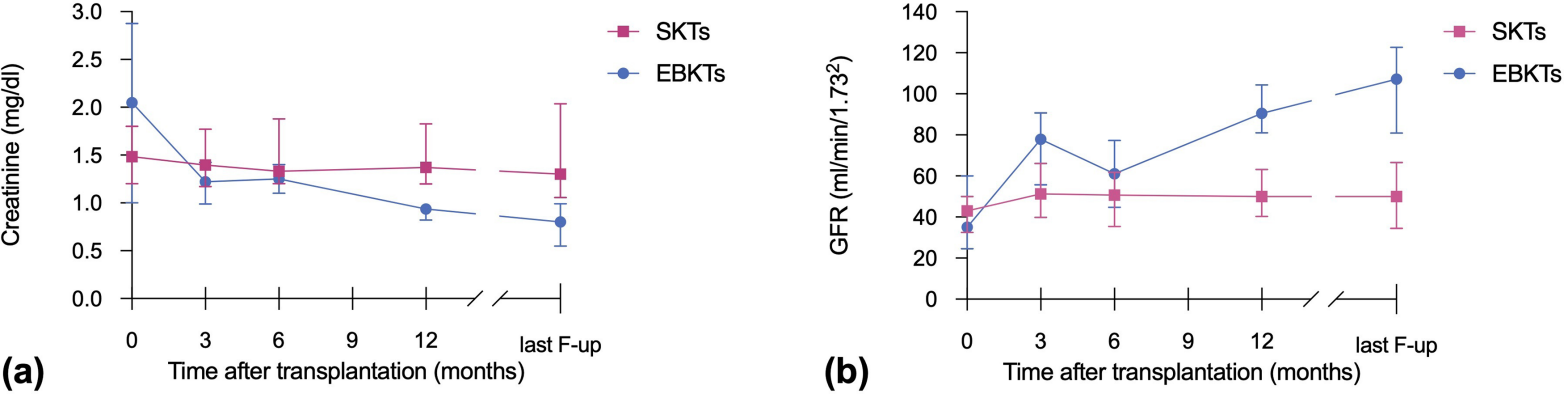
Course of **(a)** creatinine and **(b)** eGFR after SKT vs. EBKT of the PSM cohort.

Similar to the whole cohort, in the PSM subgroup analysis, median serum creatinine and median eGFR at 1 year post transplant were superior in EBKTs compared to SKT: 0.94 mg/dl and 90.5 ml/min/1.73m^2^ compared to 1.3 mg/dl and 49.9 ml/min/1.73m^2^, respectively; *p* = 0.006 for both. At last follow-up, median serum creatinine was 0.8 mg/dl compared to 1.3 mg/dl, median eGFR reached 107.2 ml/min/1.73m^2^ compared to 50.0 ml/min/1.73m^2^; *p* = 0.001 and *p* < 0.001, respectively (Figure 6).

## 4 Discussion

The major finding of this study is that, despite the higher risk for perioperative complications, in particular vascular thrombosis, EBKT recipients experience excellent 5-year patient and graft survival rates of 100%, with an eGFR at last follow up twice as high compared to SKT recipients. However, the small sample size in the EBKT group limits statistical power, necessitating cautious interpretation of these findings.

After unsatisfactory first historical reports (11, 23), EBKT has emerged as a valuable option in adult kidney transplantation. Still, surgical complications, in particular vascular and ureteral complications are feared when transplanting very small pediatric kidneys. In the literature, the majority of early graft losses are attributed to vascular complications, with a varying incidence ranging from 2% to 23% (14, 24–26). We did not observe any combined graft loss due to vascular complications in our EBKT group. In our series, two patients underwent unilateral graft nephrectomy due to arterial thrombosis—one recipient of the smallest pediatric donor kidneys in our series (donor weight 3,000 g) and one with insufficient anticoagulation in the immediate postoperative period—however, unilateral nephrectomy in our EBKT-cohort did not negatively impact long-term graft function or survival. Of note, both EBKT patients experiencing graft thrombosis in our cohort had undergone kidney re-transplantation. There is currently no data available on the outcomes of EBKTs in the setting of re-transplantation. While standard kidney re-transplantation has been shown to provide survival benefit over dialysis and achieve outcomes comparable to primary transplantation (27–29), the specific risk of retransplanting en-bloc grafts remains largely unexplored. One potential contributing factor may be the administration of higher doses of tacrolimus in retransplant recipients (30, 31). Given that EBKT is already associated with a higher incidence of vascular complications, these risks may be further amplified in re-transplant recipients.

Beyond vascular complications, concers regarding ureteral complicatiosn have also been raised in the context of EBKTs. Seizilles de Mazancourt et al. reported a 33% ureteral complication rate, including three ureteral reimplantations after necrosis of the bladder patch, yet, graft survival was 93% in their series with only one early graft loss due to venous thrombosis (25). Fananapazir et al, reported an overall rate of postoperative ureteral complication rate of 9.8%. Again, ureteral complications did not appear to negatively affect patient or graft survival (32). In our EBKT group, we did not observe any ureteral complications. We performed all ureteral anastomoses applying the Lich-Gregoire technique. In our opinion, the fear of urological complications following EBKT, should no longer discourage their acceptance as urologic complications do not adversely affect overall outcome.

Despite the higher incidence of postoperative complications and reinterventions, our data, as well as several recent studies demonstrate excellent patient and graft survival rates following EBKTs (24, 25, 32–35). Seizilles de Mazancourt et al. reported a 100% patient survival rate and 95% graft survival rate in their EBKT cohort after median 62 months of follow-up (25). Similarly, López-González et al. analyzed 42 adult recipients of pediatric en-bloc kidney transplants, reporting a graft survival rate of 83.3% after a median follow-up of 73 months. In their study, seven graft losses occurred in the immediate postoperative period, with 4 due to vascular thrombosis (24). Another study compared outcomes between EBKT and living donor kidney transplantation, demonstrating similar graft survival rates after a follow-up of up to 27 years, while eGFR was significantly higher in EBKTs recipients (33). An analysis of the UNOS/STAR data from 1988 to 2006 included 1,696 en-bloc kidney transplants from donors younger than 5 years and compared the results to solitary pediatric kidneys, SCD and ECD kidneys. EBKT demonstrated the most favorable long-term graft survival at 10 years compared to the other groups (34).

Eastment and colleagues recently compared the outcome of single vs. dual en-bloc kidney transplantation from donors aged ≤5 years. They found no difference in terms of patient and graft survival, or serum creatinine levels post transplantation (36), however, their findings are limited by differences in donor age and weight between the two groups. Of note, their study included only 5 kidneys from donors <10 kg over a span of more than 50 years, all of them transplanted en-bloc. Similarly, a recent Canadian study, published excellent outcomes with small donors, however, groups were not equally selected (35). In 2013, a direct comparison stratified by donor weight, has supported the use of EBKTs due to superior graft survival at one year compared to single kidney transplants (37).

More recently, in 2019, Suneja et al. reported good outcomes when using kidneys from donors weighing at least 10 kg, even when used as split kidneys (13). Sampaio et al. recommended single kidney transplants in donors weighing >12 kg to adult recipients in experienced centers to optimize organ utilization (38). In pediatric recipients, EBTKs were historically not used due to concerns about an increased risk of perioperative complications, limited nephron mass, hyperfiltration injury and DGF. However, several recent studies have examined the outcomes of EBKTs in pediatric recipients, showing results comparable to those of standard kidney transplantation and even living donor kidney transplantation. Notably, long-term outcomes appear to be favorable despite the higher perioperative risk (39–44). For example Kizilbash et al. reportet that EBKTs in children were associated with superior long-term graft and patient survival compared to standard deceased kidney transplants. An increased risk of early graft loss was observed only in the earliest area (1987–1979), which the authors attributed to advancements in surgical techniques and perioperative management. Moreover, EBKTs in their cohort was associated with a survival advantage compared to remaining on the waiting list (44). Currently, most kidneys from pediatric donors below 10 kg body-weight are usually retrieved en-bloc and offered to one recipient—mostly an adult (45, 46). Guidelines for clinicians to aid in decision making whether to use EBKTs or single kidney transplantation are lacking so far. Moreover, there are currently no formal restrictions preventing any center from performing EBKTs. However, given the technical complexity of the procedure, and to further optimize graft outcomes and minimize complications, it would be preferable for EBKTs to be performed only in experienced centers with expertise in pediatric transplantation and microsurgical techniques (46).

Similar to most prior studies, the recipients of EBKTs in our study were a well-selected group of patients (see selection algorithm, Figure 1). However, applying the PSM analysis confirmed the favorable outcomes observed in the overall cohort. Patient and graft survival were similar between the two groups. Importantly, also in the PSM subgroup analysis, renal function outcomes, as reflected by serum creatinine and eGFR at 1 year and at last follow-up, were superior in the EBKTs group.

Even EBKTs from donors after circulatory arrest (DCD) or acute kidney injury (AKI) demonstrate excellent outcome. Even though DCD status and donor AKI do affect early posttransplant kidney function, as indicated by the higher DGF rates and a decreased eGFR in the early posttransplant period, their use does not increase the risk for early graft loss or affect long term graft survival (47, 48). While larger, prospective studies are required to validate these observations, DCD or AKI status of small pediatric donors should not preclude the acceptance of these organs.

The KDRI was significantly higher in our EBKTs with 1.27 points compared to 1.02 points for SKT. The higher KDRI could be attributed to factors such as low donor height, weight, and age, which negatively impact the KDRI calculation. Our KDRI in EBKTs of 1.27 points would translate in an estimated three-year graft survival of around 80%, however, graft survival was 100% in our series, highlighting potential limitations in the predictive accuracy of the KDRI in this specific subpopulation, as already demonstrated by others (49, 50).

We consider careful recipient selection crucial for achieving favorable outcomes after EBKT. We allocate these kidneys to transplant candidates without significant comorbidity, particularly those without cardiovascular diseases that pose a high anesthesiologic risk (51) and who are capable of withstanding operative reinterventions. Moreover, we do not consider patients with uncontrolled hypertension as candidates for EBKT as we aimed to maintain postoperative systolic blood pressure at approximately 100 mmHG, based on the physiological systolic blood pressure ranges from 72 to 104 mmHg in infants to 85–106 mmHg in toddlers (21). Also obese patients (BMI > 30 kg/m^2^) are excluded from consideration for EBKT as these patients often suffer from multifactorial diseases, (including hypertension and cardiovascular diseases, as mentioned above) and are at higher risk of perioperative morbidity and mortality (52, 53). Postoperatively, we recommend a rigorous surveillance protocol including doppler ultrasound and clinical assessment every 6 h, with prompt intervention in case of, or even suspicion of, vascular compromise. Unfortunately, there is no uniform standard available regarding postoperative ultrasound examinations, except for the recommendation of close monitoring. In our EBKT group, the 6-hourly surveillance protocol might have rescued the kinked kidney from graft thrombosis and, in two patients, potentially prevented the progression of unilateral arterial thrombosis into the contralateral kidney, thus avoiding the loss of both grafts. Furthermore, a strict anticoagulation regime with close monitoring is recommended to carefully balance the need for thrombosis prevention with the risk of bleeding complications. We did not administer heparin before clamping, however, heparin was initiated immediately after surgery with a targeted aPTT of 40–45 s. We did not administer oral aspirin in our cohort. Unfortunately, there is a lack of evidence regarding the optimal anticoagulation regime after EBKTs.

The limitations of this study include its retrospective nature and the relatively small patient cohort. Notably, centers performing five or more EBKTs are considered high-volume centers (37). We performed 6 EBKTs within a relatively short time frame of 2.5 years, chosen to minimize variations in surgical technique or perioperative management. Generally, reports on high numbers of EBKTs procedures performed are scarce and often span a long time period, possibly resulting in a lack of standardization. The strengths of our study are that the data was retrospectively analyzed from prospectively collected, auditable medical records ensuring a high level of data completeness. Additionally, the PSM subgroup analysis enhances the robustness of our findings by reducing potential confounding factors between the groups.

In summary, kidneys from pediatric donors ≤2 years of age represent high-quality organs with the capacity to increase the donor pool. Even though EBKT of these organs pose an increased risk for perioperative complications it does not negatively impact graft and patient survival. Through careful recipient selection and meticulous postoperative monitoring, EBKTs demonstrate excellent long-term outcomes.

## Data Availability

The original contributions presented in the study are included in the article/Supplementary Material, further inquiries can be directed to the corresponding author.
